# Supplementary material for: Deep intravital brain tumor imaging enabled by tailored three-photon microscopy and analysis
Source: Nat Commun. 2024 Sep 10;15:7383. doi: 10.1038/s41467-024-51432-4 (PMC11387418; doi:10.1038/s41467-024-51432-4)
Supplement: Supplementary file 1 — Supplementary Information [file 41467_2024_51432_MOESM1_ESM.pdf]

# Supplementary information

## Deep intravital brain tumor imaging enabled by tailored three-photon microscopy and analysis

Marc Cicero Schubert[1,2#], Stella Judith Soyka[1,2#], Amr Tamimi[3#], Emanuel Maus[3], Julian Schroers[1,4], Niklas Wißmann[1,2], Ekin Reyhan[1,5], Svenja Kristin Tetzlaff[1,2], Yvonne Yang[1,5], Robert Denninger[1,2], Robin Peretzke[6], Carlo Beretta[2], Michael Drumm[7], Alina Heuer[1], Verena Buchert[1], Alicia Steffens[7], Jordain Walshon[7], Kathleen McCortney[7], Sabine Heiland[8], Martin Bendszus[8], Peter Neher[6,9,10], Anna Golebiewska[11], Wolfgang Wick[1,5], Frank Winkler[1,5], Michael O. Breckwoldt[8], Anna Kreshuk[3], Thomas Kuner[2], Craig Horbinski[7,12], Felix Tobias Kurz[4,8,13], Robert Prevedel[3,14,15,16,17], Varun Venkataramani[1,2,3,5]

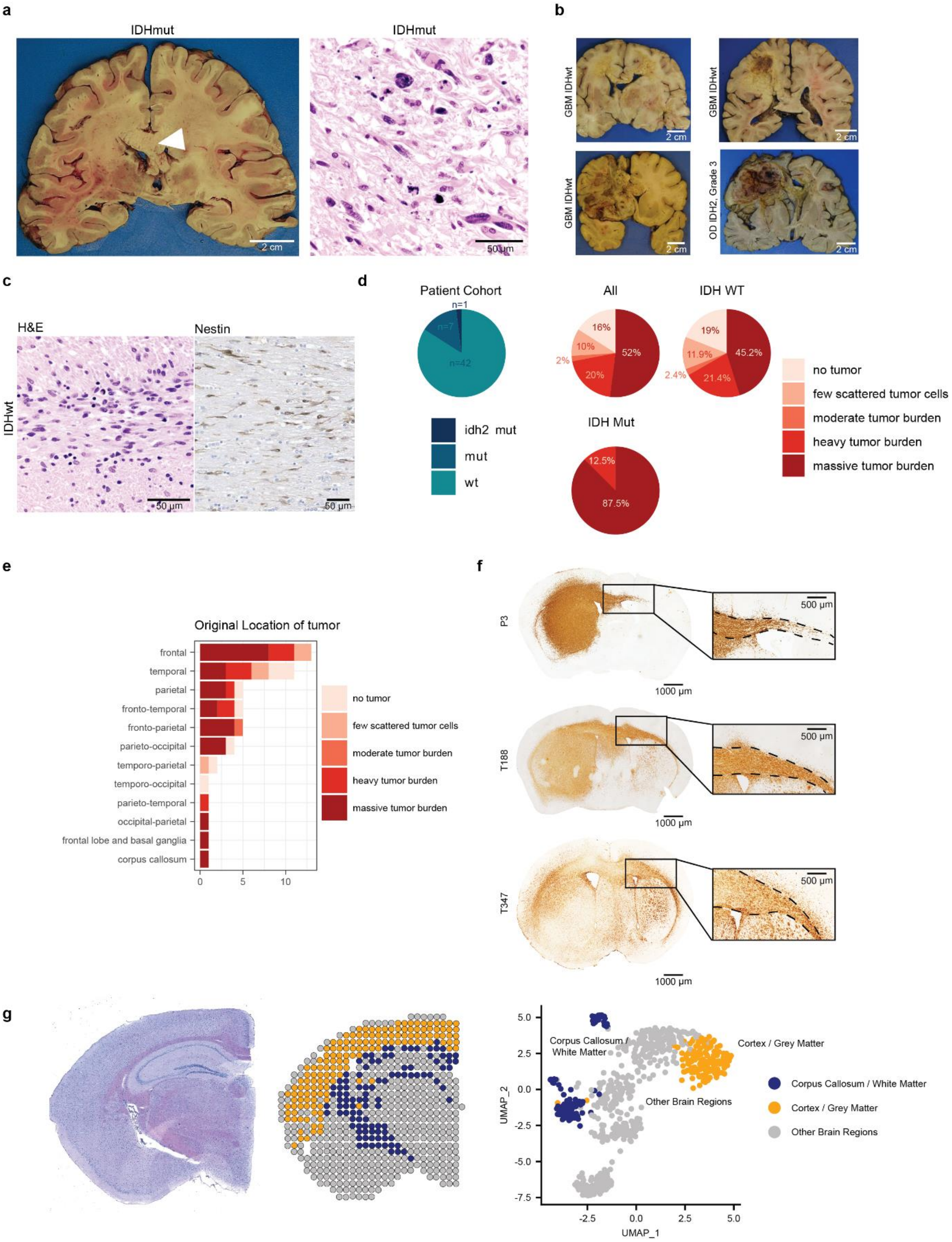


**Suppl. Fig. 1 Colonization of CC as a hallmark of glioma growth patterns. a,** Human autopsy from a patient with an IDH-mutant glioma and the associated histological sample. Arrowhead shows tumor infiltration in the CC. **b,** Coronal sections of brain tumor patients with IDH-WT glioblastoma and oligodendroglioma, WHO grade III showed streaks of yellow necrosis through the CC. **c,** On microscopic examination, sections of the CC showed

extensive tumor infiltration on H&E (left) and in immunohistochemistry for nestin (right). On the left, histological sample from autopsy section in Fig 1a is shown. **d,** Analysis of patient cohort regarding IDH mutation (n = 50 patients). **e,** Analysis of original location of tumor and their CC infiltration. The number of patient samples is displayed on the x-axis. **f,** Examples of three different PDX models stained with an antibody against nestin for the visualization of GBMCs and subsequent DAB precipitation (from top to bottom: P3, T0188, T347) illustrating infiltration into the CC. Infiltration of the CC can be seen on the inset. **g,** Spatial transcriptomic slice based on the Molecular Atlas of the Mouse Brain (left), and their molecular heterogeneity (right), visualized as UMAP (n = 639 spots). Source data are provided as a Source Data file.

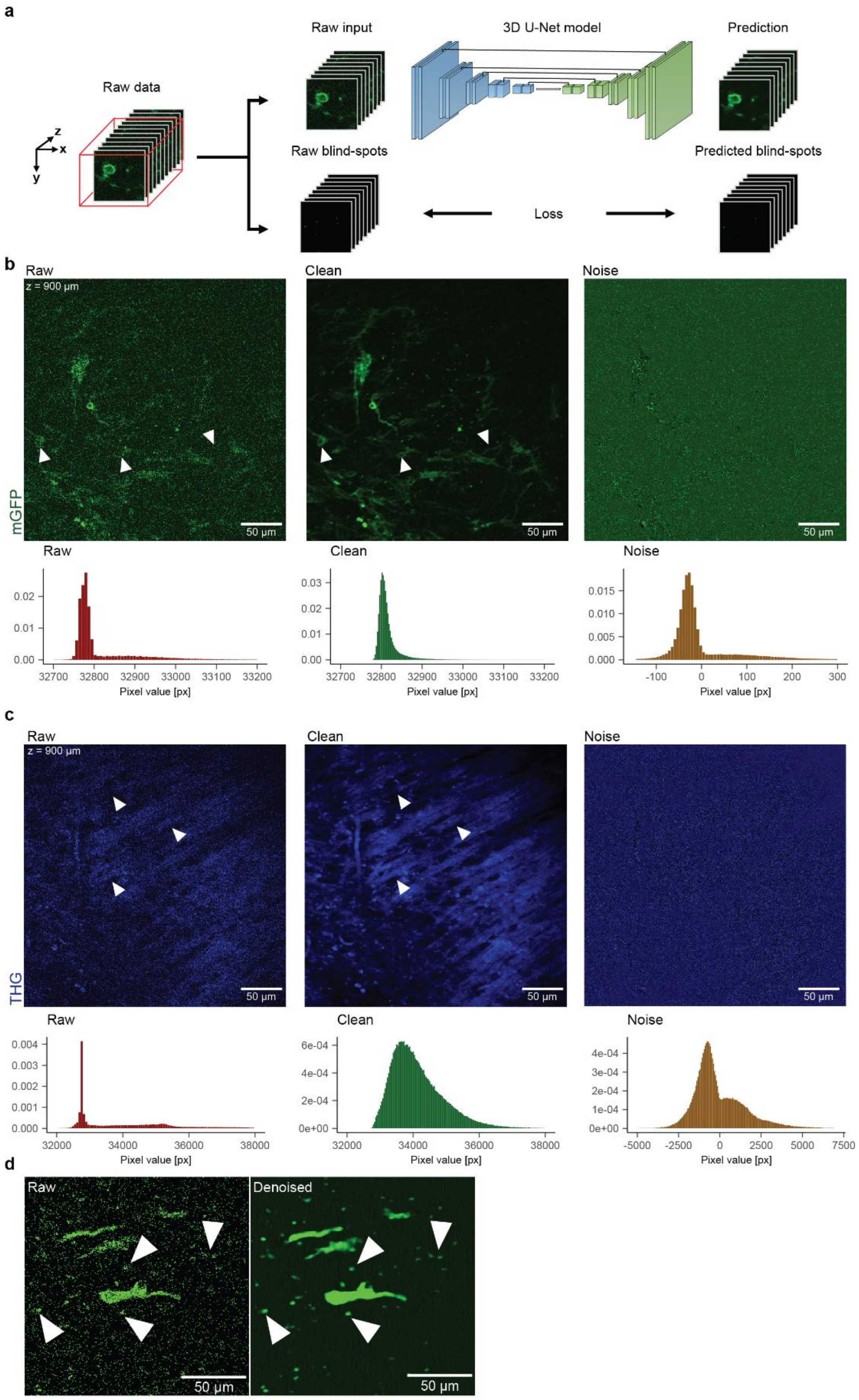


**Suppl. Fig. 2 Training procedure of the three-dimensional 3PM-Noise2Void and the noise characteristics of the 3PM two-channel recording. a,** Schematic representation of the 3PM-Noise2Void during training. Before the raw data is blind spotted and used for training, the three-dimensional 3PM data is sliced into smaller patches. The 3D U-net model is trained to minimize the discrepancy between the raw and predicted blind-spots. **b,** Clean

and noise components of the 3PM mGFP signal with low SNR, illustrating the detector and shot noise and their narrow distribution. The clean images were estimated by averaging 60 raw images. The arrowheads point to exemplary GBMC somata and TMs. **c,** Clean and noise components of the 3PM THG signal with high SNR, showing the effects of the same noise sources and their broader distribution. The arrow-heads point to exemplary myelin fibers. **d,** Pre- and post-denoising of exemplary region (as shown in Fig. 5a) showing that non- cellular fluorescent dots are no artefact of denoising but can already be observed in raw data. Source data are provided as a Source Data file.

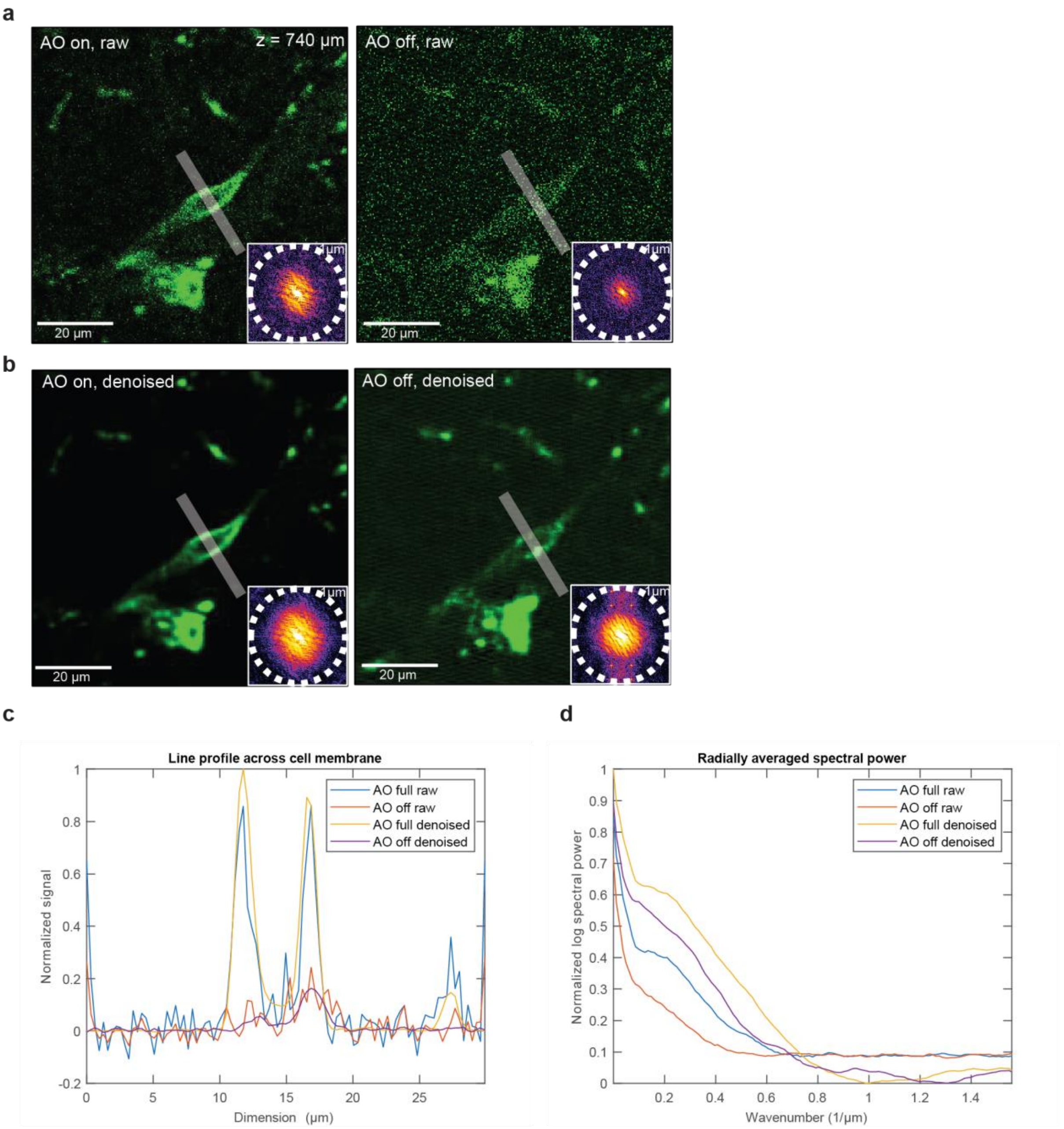


**Suppl. Fig. 3 Effects of combining adaptive optics with AI-based denoising. a,** Exemplary mGFP labeled GBMC imaged with (left) and without (right) AO optimization. **b,** The same panels are shown after denoising. The gray line indicates the line segment averaged over to produce the line profiles, showing the effect of uncorrected optical aberration on the visibility of fine cellular structure. The inset on each panel in **a** and **b** show the frequency domain power spectrum of the image. **c,** Line profile comparisons showing intensity enhancement of the AO images. **d,** The averaged radial profile of the frequency maps are shown, allowing easier estimation of the respective frequency cut-offs. We note that the elevated spectral power of the AO off denoised image compared to the non-denoised case is likely due to the enhanced contrast and thus effectively sharper image features. Nevertheless, the overall lower signal of AO off leads to qualitatively lower image quality, with less fine image features observable (e.g. tumor microtubes).

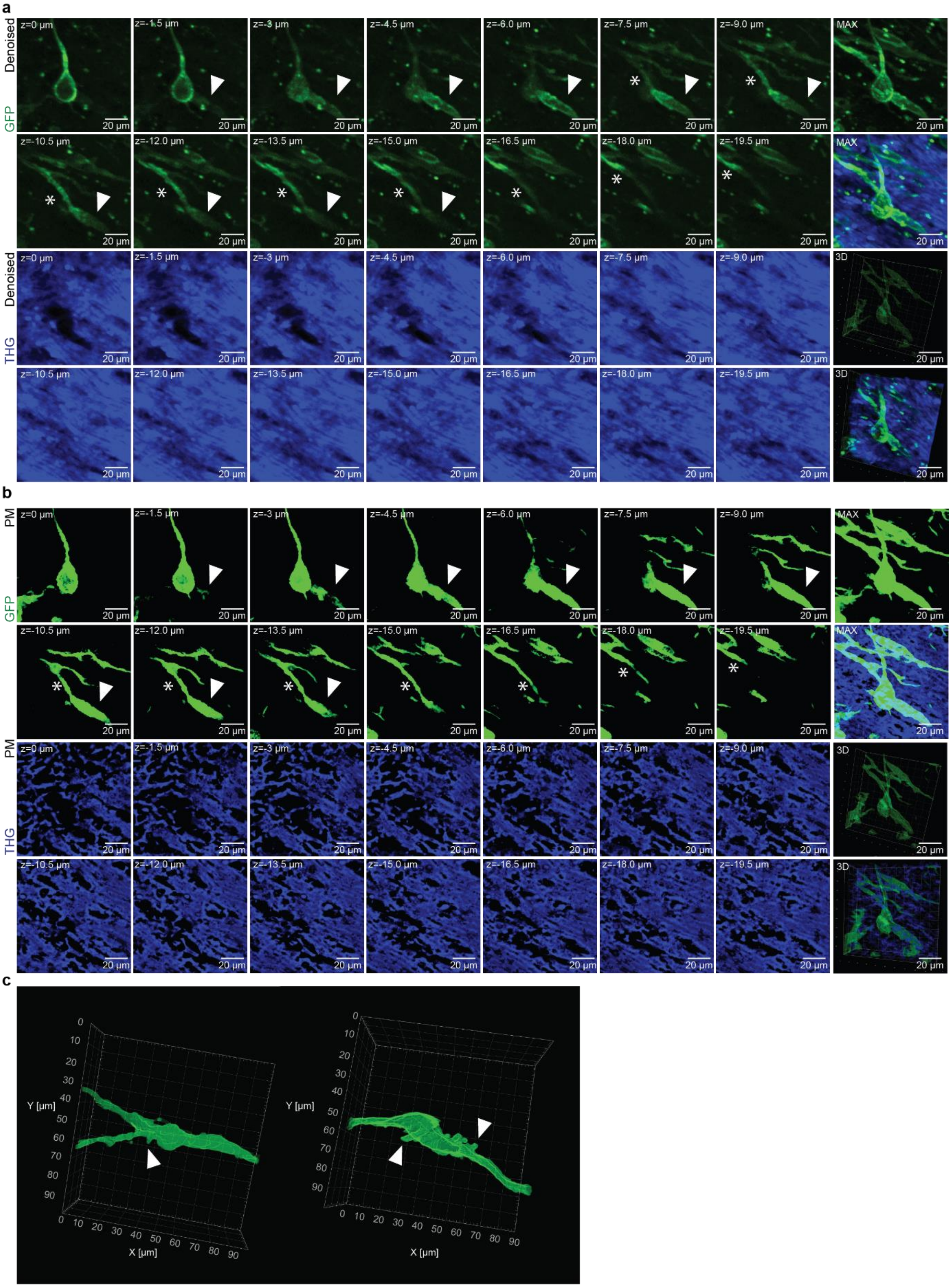


**Suppl. Fig. 4 Exemplary cells across z-slices. a, and b,** Cells are shown across z-slices, as MIPs in both channels and in both pre-segmentation and post-segmentation as probability maps. **c,** Close up 3D renderings of

two S24 GBMCs based on probability maps. The arrowheads point at small processes. Gamma values were adjusted for visualization in c.

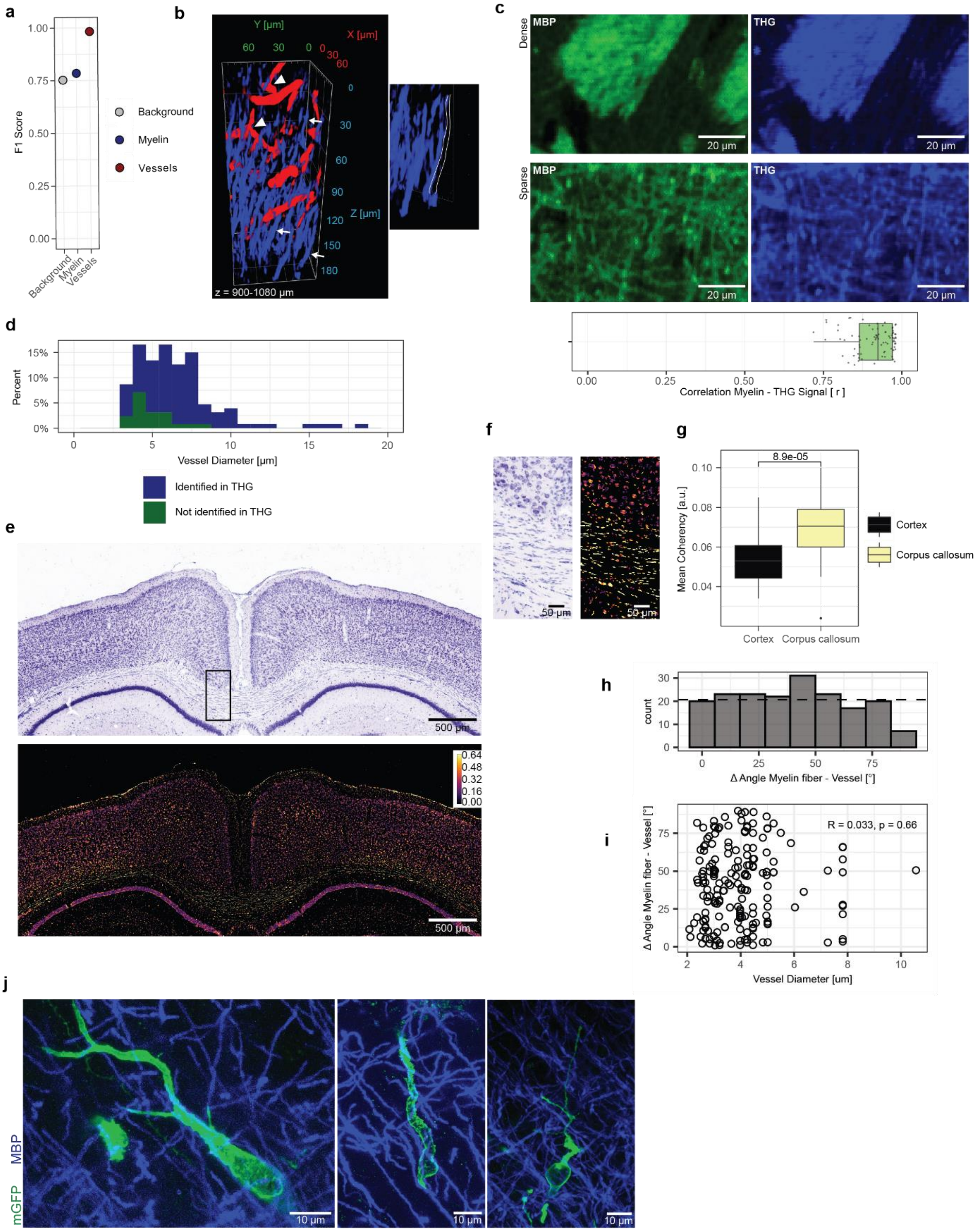


**Suppl. Fig. 5 THG signal analysis and validation of machine learning-based classification of the THG signal.**

**a,** F1 score of the machine learning based-classification as compared to the human annotated ground truth signal classification. **b,** 3D rendering of blood vessels (red) and myelinated fibers (blue) in deep cortical regions, classified

using customized machine learning. Arrowheads indicate blood vessels; arrows indicate vertical myelin fibers. Myelinated fibers in the deep cortex going to the CC can be seen on the right. Gamma values were adjusted for 3D visualization. **c,** Top rows: Immunofluorescence staining of MBP (myelin basic protein, left) and THG signal on the right in two regions differing in the density of myelin fibers. Bottom row: Correlation analysis of myelin and THG signal in pixel intensity (n = 8 different regions, each n = 10 ROIs)**.** **d,** Histogram of measured blood vessels based on their diameter and colored based on their identification from THG signal in tumor-infiltrated regions (blue: visible with FITC and in THG signal, green: visible only with FITC, n = 127 vessels). **e**, Exemplary brain slide from Allen Brain Atlas, Nissl Staining (top). Bottom: Coherency map, color-coded based on coherency measure. Multiplication of the segmentation of the Nissl-Staining multiplied by coherency-measurement output is shown. Black Rectangle shows region for the zoom-in in **f**. **f**, Zoom-in of region marked in **e**, with histology slide (left) and coherency map (right). **g**, Comparison of coherency in n = 30 vs n = 30 regions in the CC vs the cortex, identified in n = 3 different slides from the Allen Brain Atlas. **h**, Histogram of angles between Myelin fibers and close-by vascular sections, dashed line indicates continuous uniform distribution. **i,** Angle between myelin fibers depending on vessel diameter, n = 186 vessel segments from n = 86 vessels. **j,** Confocal microscopy images in the cortex showing GBM cells (green) and close-by myelin fibers labeled with MBP staining (blue). Source data are provided as a Source Data file.

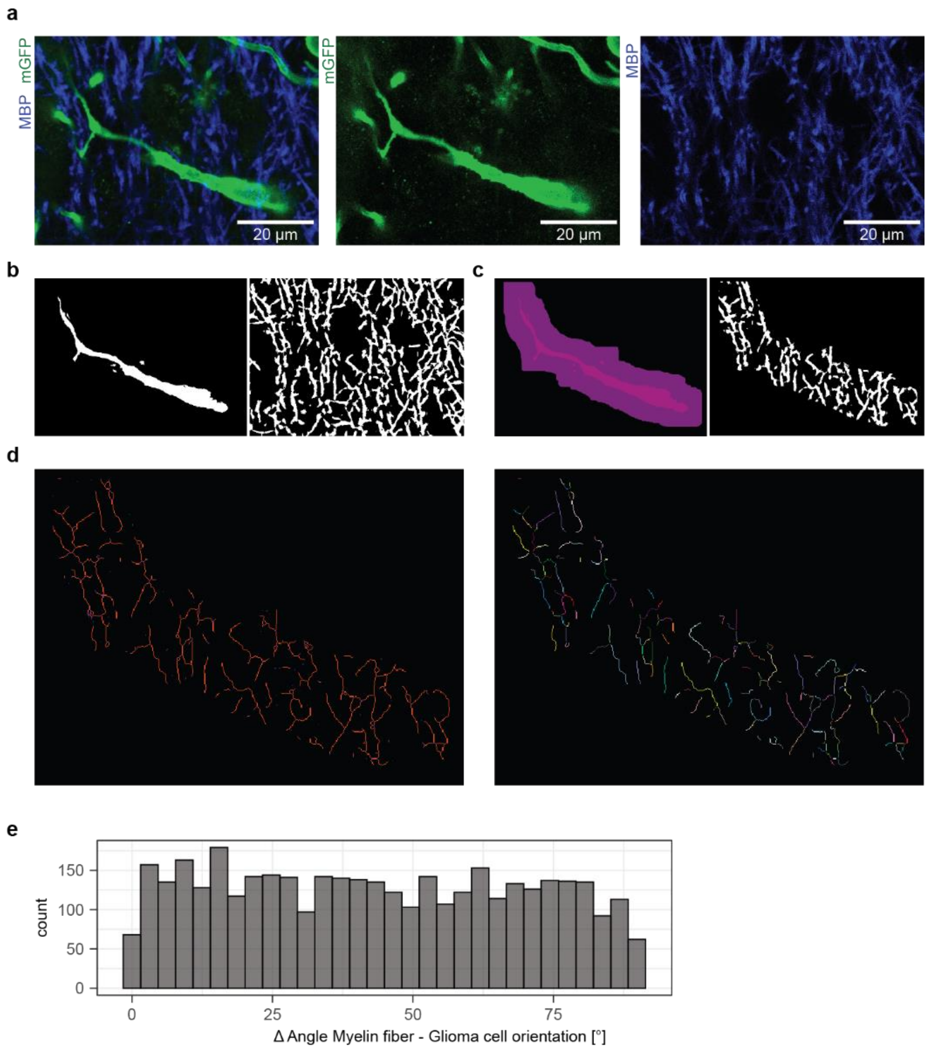


**Suppl. Fig. 6 Angle analysis between myelin fibers and glioma cells in the cortex. a**, Exemplary GBMC in the cortex within myelin network, composite (left), GBMC (middle) and myelin fibers (right). **b**, Segmentation of GBMC (left) and myelin fibers (right) **c**, Dilated mask of GBMC by 5 µm (left) and mask multiplied by myelin fiber mask (right). **d**, Skeletonization of myelin fibers before and after removal of branching points to split merged fibers into individual branches and removal of branches with 4 or less pixels (left and right, respectively). Right: Individual branches colored in unique colors. **e**, Distribution of angle between skeleton branches and major axis of GBMCs, quantified in n = 45 cells and n = 3823 associated myelin fibers, in n = 3 different mice. Source data are provided as a Source Data file.

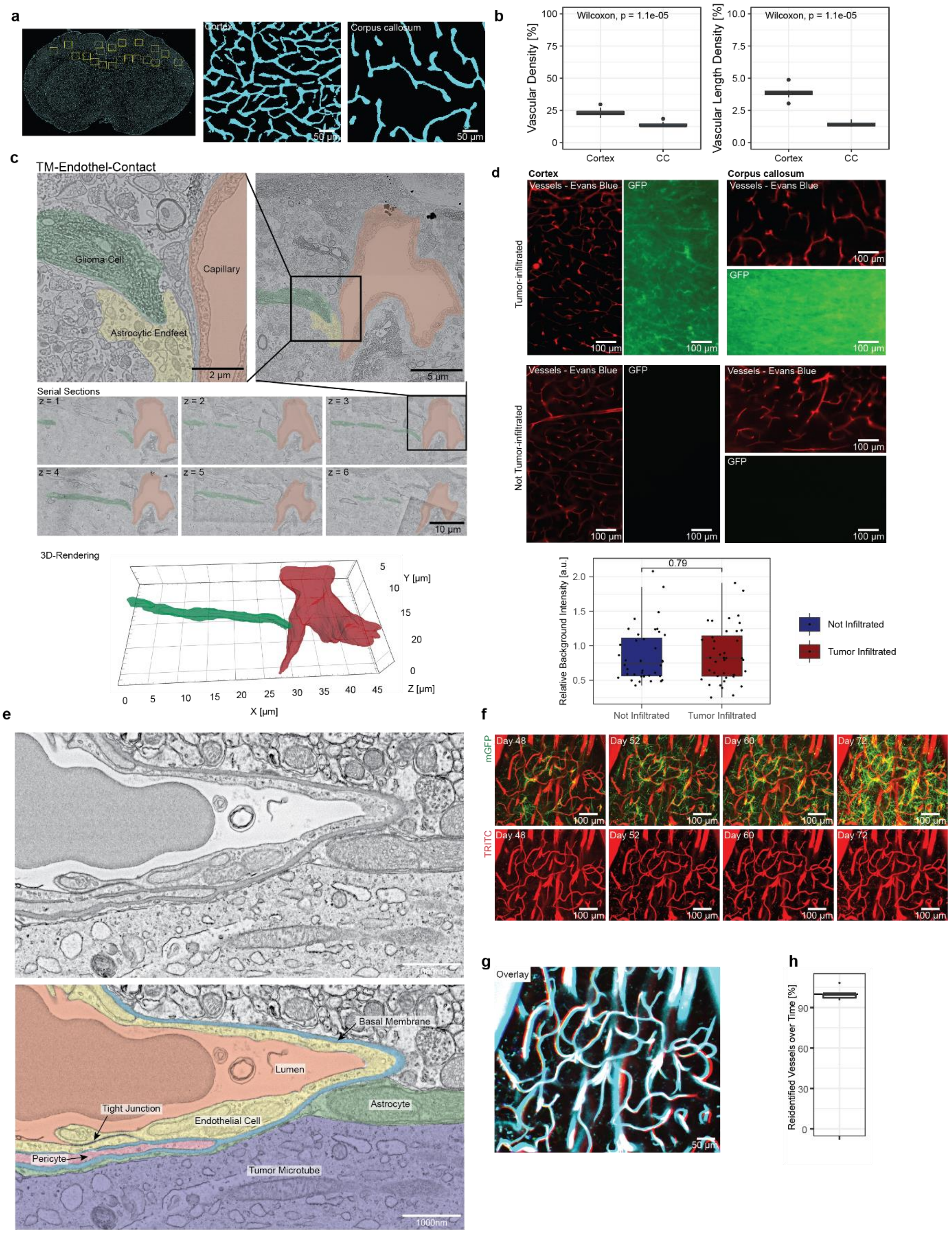


**Suppl. Fig. 7 Vascular density, vessel-guided glioblastoma translocation and integrity of blood brain barrier. a,** Vascular density in cortex compared to CC. Overview of mouse brain section, analyzed regions in the cortex and CC (yellow, left), exemplary regions in the cortex and CC (right). **b,** Vascular density (percentage of vessel area of total area), and Vascular length density (percentage of skeletonized vessel area of total area) in n

= 20 regions of interests (n = 10 each, in CC and the cortex) shown in **a**. **c,** Top row: Scanning electron microscopy (SEM) image of an orthogonal contact of a TM (green) on a blood vessel (red). Astrocytic endfeet colored in yellow, cell type based on ultrastructural features. Zoom-in on the left side ( n = 13 GBCs from n = 5 PDX mice). DAB-precipitate at anti-nestin-stainings. Middle row: Serial section of SEM images showing the GBC-vessel-contact. Bottom row: 3D rendering of the TM-tip on the capillary using manual segmentation of 20 serial 2D-electron microscopy (EM)-sections. Gamma values were adjusted for 3D visualization. **d**, Vascular integrity in cortex and CC in tumor infiltrated (top) and non-tumor infiltrated regions (middle)**.** Evans Blue shown in red, tumor cell tissue shown in green. Bottom: Relative intensity of background in n = 40 infiltrated brain regions vs n = 40 non-infiltrated regions (n = 4 mice). **e**, EM image of a TM in close proximity to the blood-brain-barrier (BBB). The integrity of the BBB is demonstrated by an intact, continuous basement membrane and intact tight junctions. Furthermore, a continuous astrocyte (green overlay) and a pericyte (pink overlay) can be seen. **f**, Microscopic blood vessel architecture over time at multiple timepoints during early brain tumor progression. In vivo images of GBMCs (green) and vessels (red) are shown. Intravenous injection of TRITC- dextran was used to visualize vessels for the angiogram. **g**, Overlay of the vessel channel of all four timepoints in **f**. **h,** Percentage of vessels observed at time point 2 compared to time point 1 (n = 6 regions in n = 3 mice). Source data are provided as a Source Data file.

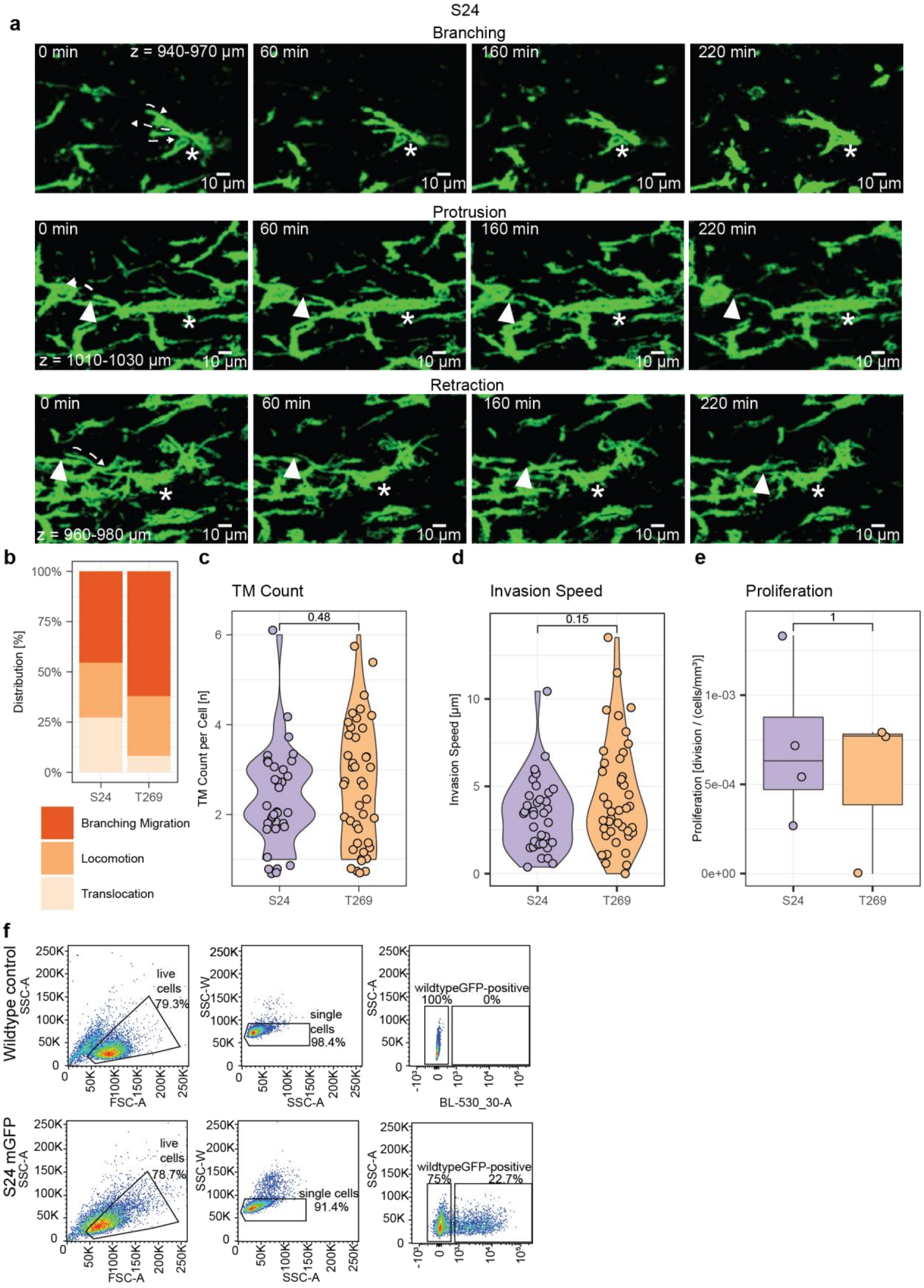


**Suppl. Fig. 8 Tumor microtube dynamics in the CC in further PDX models. a**, MIP time-lapse images showing TMs that use branching, protrusion, and retraction in the CC in the PDX model S24 (n = 3 S24 PDX). Asterisks point at the somata, the dashed arrows indicate the direction of the tumor microtube dynamic. Arrowheads point

at the tip of the TM of interest. Data are shown as probability maps and are post-processed with the ‘‘smooth” function in Fiji. **b,** Distribution of invasion phenotypes compared between S24 and T269 PDX model (n = 33 and n = 37 cells). **c,** TM count per cell compared between PDX models (n = 34 cells in S24 and n = 43 cells in T269, in n = 6 mice) **d,** Invasion speed per hour compared between PDX models (n = 36 cells in S24 and n = 42 in T269, in n = 6 mice), **e,** Proliferation between PDX models, quantified as cell divisions per cell density (n = 4 experiments in S24 and n = 3 experiments in T269, quantified as divisions / (cells/mm$^3$). **f**, Upper row: representative gating strategy for wildtype control. Lower row: gating strategies for S24mGFP cells. Source data are provided as a Source Data file.

a

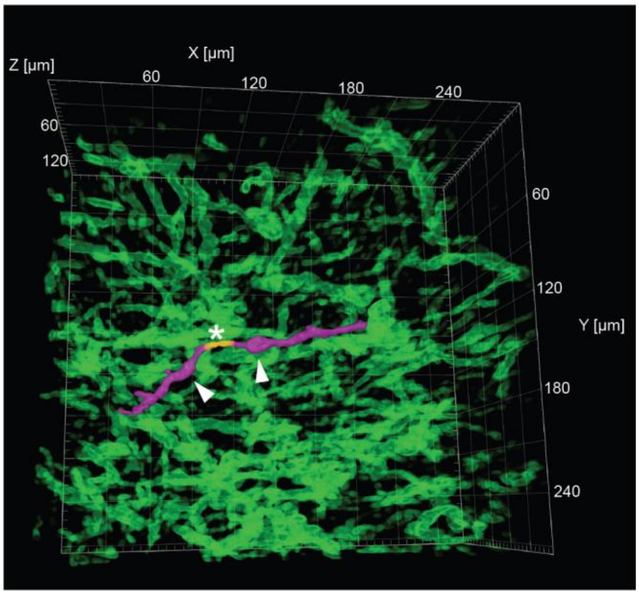


b

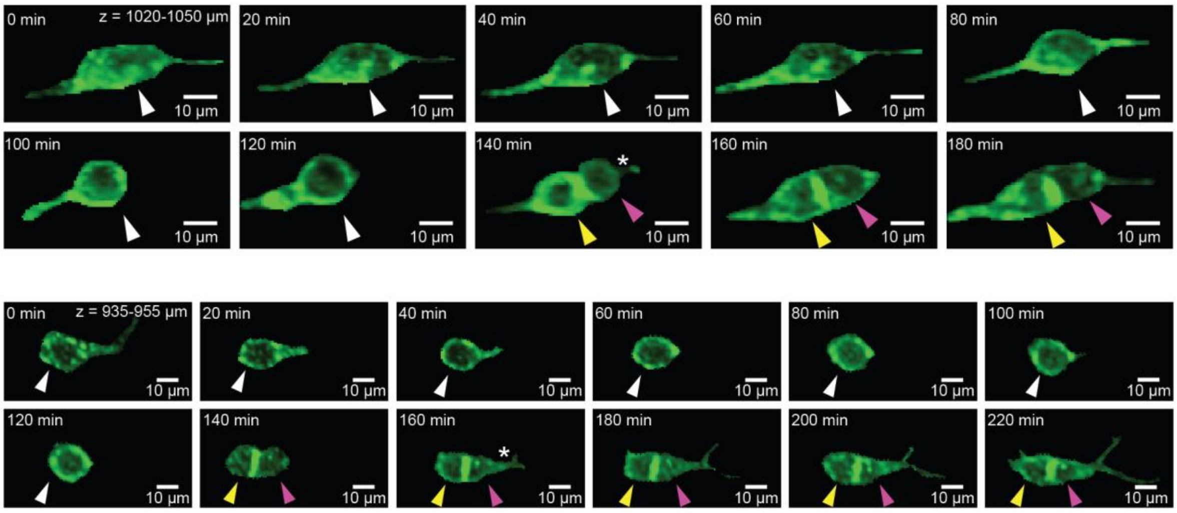


**Suppl. Fig. 9 Time-lapse of tumor cell proliferation in the CC. a,** Rendering of tumor cell network. An exemplary tumor-tumor cell connection is visualized (yellow, asterisk) that connects two tumor cells (purple, arrowhead). Gamma values were adjusted for 3D visualization. **b,** MIP time-lapse imaging of tumor cell proliferation in the CC. White arrowhead: GBMC before cell division. Yellow and purple arrowhead: Daughter GBMCs after cell division. The asterisk points at a newly grown TM. Top: S24 PDX model, bottom: T269 PDX model. Post-processed with denoising and “clear outside” function in ImageJ/Fiji.

**a**

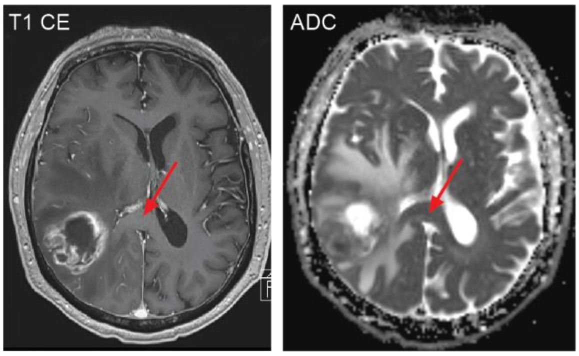


**b**

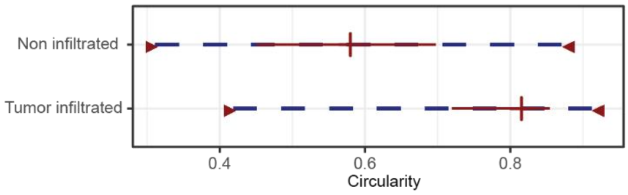


**Suppl. Fig. 10 CC involvement in further GBM patient shown in MRI scans. a,** Additional representative MRI scan of a GBM patient showing CC (red arrows) involvement in Gadolinium- contrast enhanced T1w and ADC images. **b,** Ranges of occurring circularities in the measured cells in Fig. 8g, red triangles indicate minimum and maximum values, red vertical line: median, red horizontal line: quartiles, dashed blue line: range.

**Supplementary Note**

**Deep3P imaging considerations and workflow**

Due to the limits on pulse energy and average power that can be safely delivered[1] to the focal volume during imaging, it is important to have optimized window placement and properties as well as a refined acquisition plan in order to find and characterize volumes of interest. In case that no labeled cells are found initially, it is not clear whether they are absent, or the signal to noise is simply too poor to resolve them. Conducting the exploration while exceeding recommended laser power levels may result in damage to the brain regions explored as well as bleaching the fluorescent label in those volumes. Having an additional structure labeled, or in the case of this work, a label-free signal like THG, greatly aids in ensuring that absence of labeled cells is not mistaken for poor signal to noise.

Poor signal to noise performance can result from microscope issues (uncompressed pulses, clipped or distorted beam profiles, misalignment etc.), window issues (optical quality, curvature, glue/dirt obscuration, inflammation, bad placement etc.), or from brain structures obscuring the infiltrated areas (large blood vessels or other absorbing structures). All three sources can also produce a distorted PSF due to strong aberration, resulting in low SNR for 3PM, and which in extreme cases cannot be fully mitigated by AO optimization.

It is therefore critical that the cranial window is of the highest optical quality, placed over a region of the brain ensuring wide and deep access to the most accessible white matter structures, and that the glue is both secure and not obscuring or contaminating the imaging area. The longitudinal 2PM imaging of glioblastoma in the mouse brain is helpful in tracking the progress of tumor progression. Subsequently, the goal is then to optimize access to as much white matter as possible that yields desirable SNR properties. The inhomogeneous illumination in cortex and the corpus callosum also means that simply turning up the laser power is not advisable. One hemisphere can be privileged for wider or deeper access by moving the window off-center from the sinus midline towards the target hemisphere. Therefore, an imaging experiment proceeds with identifying the accessible patches, then

acquiring volumes to screen for labeled cell infiltration. This proceeds most easily by locating the depth of the window surface, the lateral position of the sinus, and then moving 1200 µm from the sinus laterally to a relatively unobstructed cone of light. Proceeding to roughly 900 µm depth (ensure the objective correction collar is set to this depth), the power can be increased gradually to approximately 80% of the threshold power expected for that depth and brain anatomy, as estimated by taking the effective attenuation length of brain tissue into account. As the power is brought up and travels lengthwise with the objective, THG signal from the white matter will increase dramatically when an accessible patch is encountered. Once a path is found, higher resolution characterization reveals the depth and thickness of the CC, identified by visible fiber alignment. Once the desired imaging volume is thus identified, labeled cells can either be directly screened, or an AO optimization on either the white matter THG or on any labeled cells can be performed. Optimizing the AO before intensive characterization helps ensure that the minimum necessary power is used, minimizing light exposure and enabling a four hour time-lapse experiment to be conducted without photodamage or significant bleaching.

## Supplementary Methods

### Pre 3PM- workflow

Deep brain tumor imaging requires optimal conditions regarding pre- acquisition parameters. We implemented an approach for chronic cranial window implantation that allows a better accessibility of the corpus callosum by transplanting the window asymmetrically above the superior sagittal sinus (Suppl. Fig. 11).

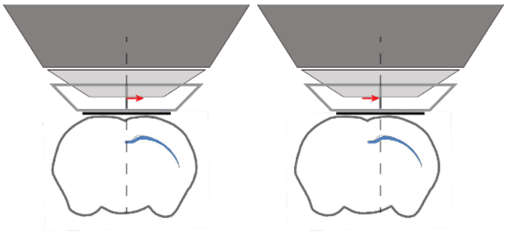

**Suppl. Fig. 11 Cranial window implantation.** Schematic of off centered cranial window implantation approach with better imaging accessibility of corpus callosum (blue).

After tumor cell injection into the cortex we screened for tumor growth regularly with 2PM, considering the advantage of faster imaging speed. Tumor growth was assessed regarding dissemination and density of tumor cells. 3P imaging was initiated when tumor cells could be detected up to a z-depth of approximately 700 µm (Suppl. Fig 12). We additionally screened the contralateral hemisphere, where no tumor cells had been injected prior as tumor cell dissemination in the contralateral hemisphere indicates infiltration into the CC. An area of interest is then chosen, taking into account probable accessibility of CC, probable tumor cell density and presence of superficial landmarks for recognition (Suppl. Fig. 13).

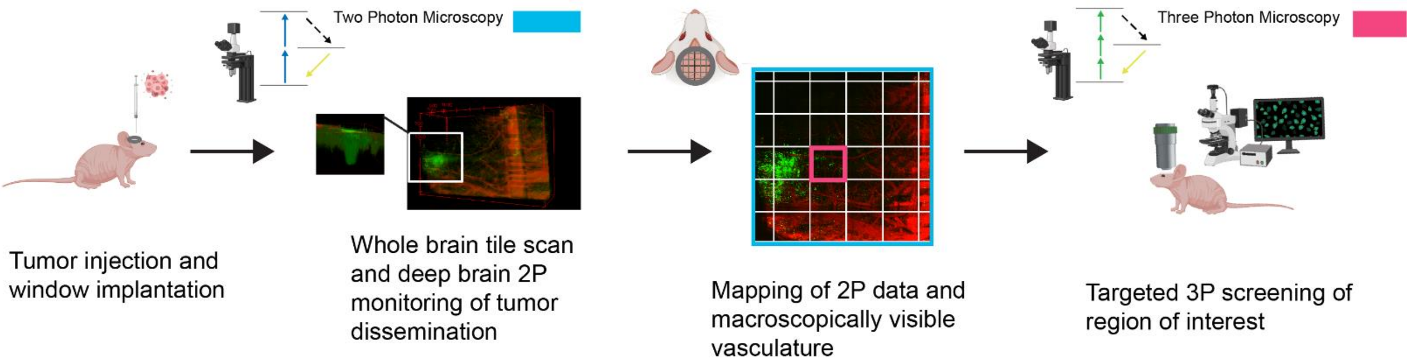


**Suppl. Fig. 12 Pre-3P workflow.** Workflow showing 2PM screening and mapping of tile scans as a preparation for 3PM. Created with BioRender.com released under a Creative Commons Attribution-NonCommercial-NoDerivs 4.0 International license (https://creativecommons.org/licenses/by-nc-nd/4.0/deed.en).

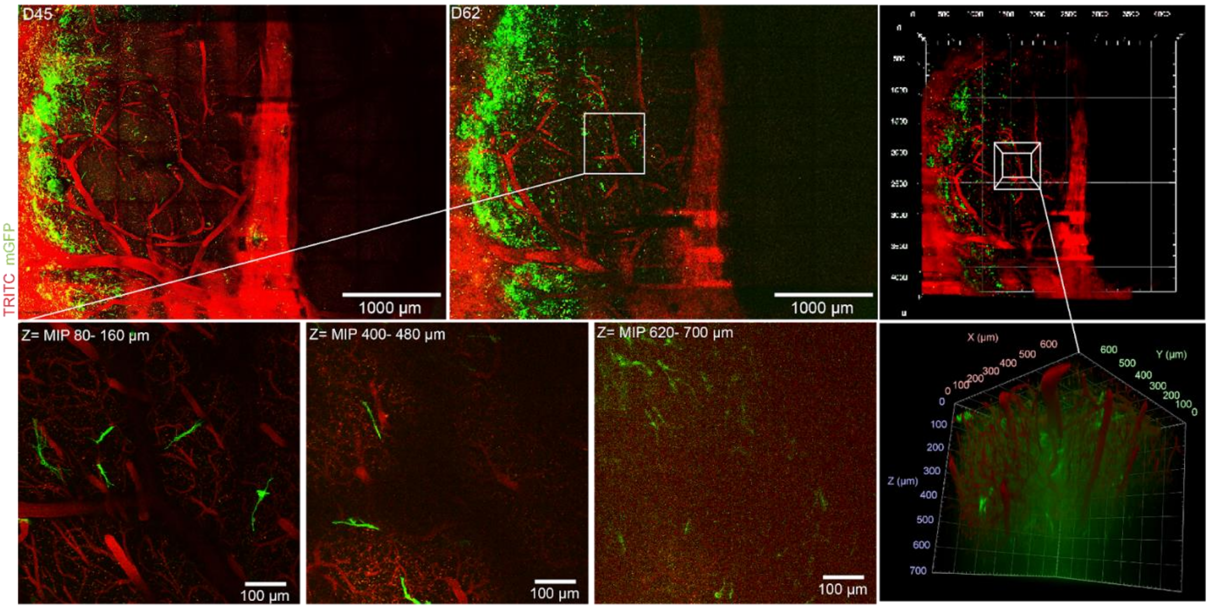


**Suppl. Fig. 13 Reidentification of vasculature.** Upper row (left and middle): MIP of 2PM tile scan showing the vasculature and tumor infiltration within the hemisphere ipsilateral to tumor cell injection at day 45 and day 62 post surgery. Lower row: Tumor cell infiltration on day 62 post surgery at different imaging depths within the mouse cortex up to a depth of 700 µm. Right: 3D Stack showing tumor infiltration within the cortex at day 62 post surgery.

To facilitate 2P-3P- correlation, we mapped the macroscopically visible vasculature to the 2PM- tile scans, which later allows faster recognition of the area of interest (Suppl. Fig. 14).

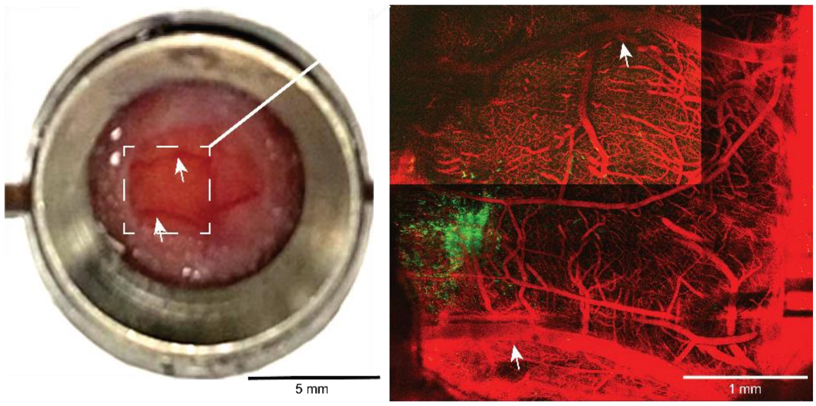


**Suppl. Fig. 14 Macroscopic view of chronic cranial window**. Chronic cranial window revealing the superficial vasculature of the mouse brain (left). On the right, the MIP of the corresponding 2PM- tile scan is shown. Arrows point at corresponding vessels in both images. Contrast adaptions have been applied to parts of the image on the right for better depiction of the vessels of interest.

## Supplementary References

1 Yildirim, M., Sugihara, H., So, P. T. C. & Sur, M. Functional imaging of visual cortical layers and subplate in awake mice with optimized three-photon microscopy. *Nature communications* **10**, doi:ARTN 177 10.1038/s41467-018-08179-6 (2019).
