# Supplementary material for: Deep intravital brain tumor imaging enabled by tailored three-photon microscopy and analysis
Source: Nat Commun. 2024 Sep 10;15:7383. doi: 10.1038/s41467-024-51432-4 (PMC11387418; doi:10.1038/s41467-024-51432-4)
Supplement: Supplementary file 2 — Description of Additional Supplementary Files [file 41467_2024_51432_MOESM2_ESM.pdf]

Description of Additional Supplementary Files

Supplementary Data 1: Data of glioma patients analyzed for macroscopic and microscopic infiltration of the corpus callosum.

Supplementary Data 2: Molecular characterization of patient-derived glioblastoma cell lines and PDOX models used in this study.

Supplementary Data 3: Acquisition parameters for high resolution *in vivo* imaging at different depth shown in this work.

Supplementary Movie 1: A three-dimensional rendering of a patient-derived glioblastoma xenograft model (S24) in cortex and corpus callosum with 3PM. Visualization of glioblastoma cells (green), blood vessels (red) and the corpus callosum (blue).

Supplementary Movie 2: A three-dimensional rendering of the THG signal in deep cortex layers. The signal was classified with customized machine learning into vessel (red) and myelin fibers (blue). 3PM was performed in the patient-derived xenograft model S24. Imaging depth was between 400-700 µm.

Supplementary Movie 3: 3D rendering of near-diffraction limited imaging of glioblastoma, blood vessels and white matter tracts in the corpus callosum. Small processes of the glioblastoma cell can be seen. 3PM was performed in the patient-derived xenograft model T269. Imaging depth was between 840-920 µm.

Supplementary Movie 4: Three neuronal-like cellular mechanisms of brain tumor invasion in the corpus callosum. 4D time-lapse imaging examples of branching migration, locomotion and translocation with Deep3P. Imaging depth was at 990-1020 µm.

Supplementary Movie 5: Tumor-tumor networks in white matter tracts. The S24 PDX model is shown. Glioblastoma cells are depicted in green, white matter tracts in blue and blood vessel signal in red. Imaging depth was between 900-1060 µm.

Supplementary Movie 6: Glioblastoma cell division in the corpus callosum, followed over 180 minutes. The S24 PDX model is shown. Cell is colored green before division, two daughter cells after division are colored in yellow and purple. Imaging depth was at 985-1010 µm.
