# Supplementary material for: Deep intravital brain tumor imaging enabled by tailored three-photon microscopy and analysis
Source: Nat Commun. 2024 Sep 10;15:7383. doi: 10.1038/s41467-024-51432-4 (PMC11387418; doi:10.1038/s41467-024-51432-4)
Supplement: Supplementary file 12 — Reporting Summary [file 41467_2024_51432_MOESM12_ESM.pdf]

nature portfolio

Corresponding author(s): Robert Prevedel & Varun Venkataramani

Last updated by author(s): 5/18/2024

Reporting Summary

Nature Portfolio wishes to improve the reproducibility of the work that we publish. This form provides structure for consistency and transparency in reporting. For further information on Nature Portfolio policies, see our Editorial Policies and the Editorial Policy Checklist.

Statistics

For all statistical analyses, confirm that the following items are present in the figure legend, table legend, main text, or Methods section.

n/a	Confirmed	
☐	☒	The exact sample size (n) for each experimental group/condition, given as a discrete number and unit of measurement
☐	☒	A statement on whether measurements were taken from distinct samples or whether the same sample was measured repeatedly
☐	☒	The statistical test(s) used AND whether they are one- or two-sided<br>*Only common tests should be described solely by name; describe more complex techniques in the Methods section.*
☒	☐	A description of all covariates tested
☐	☒	A description of any assumptions or corrections, such as tests of normality and adjustment for multiple comparisons
☐	☒	A full description of the statistical parameters including central tendency (e.g. means) or other basic estimates (e.g. regression coefficient) AND variation (e.g. standard deviation) or associated estimates of uncertainty (e.g. confidence intervals)
☐	☒	For null hypothesis testing, the test statistic (e.g. F, t, r) with confidence intervals, effect sizes, degrees of freedom and P value noted<br>*Give P values as exact values whenever suitable.*
☐	☒	For Bayesian analysis, information on the choice of priors and Markov chain Monte Carlo settings
☒	☐	For hierarchical and complex designs, identification of the appropriate level for tests and full reporting of outcomes
☒	☐	Estimates of effect sizes (e.g. Cohen's d, Pearson's r), indicating how they were calculated

Our web collection on statistics for biologists contains articles on many of the points above.

Software and code

Policy information about availability of computer code

Data collection	ImageJ/Fiji Schneider et al., 2012: https://imagej.nih.gov/ij/<br>Imspector<br>Matlab<br>NIS-Elements AR Analysis 5.30.01 64-bit Nikon https://www.microscope.healthcare.nikon.com/products/<br>Ilastik 1.3.3 , Berg et al., 2019 https://www.ilastik.org/development.html<br>Arivis Vision4D 3.5.0 arivis AG, Munich, Germany https://imaging.arivis.com/en/imaging-science/arivis-vision4d<br>R Studio 1.4 RStudio Team, 2020 N
Data analysis	For data analysis, custom code was written in Fiji Macro Language, Python and R. The indirect AO routines developed for integration into ScanImage32 are available at [https://github.com/prevedel-lab/AO.git].<br>AI denoising code is available on GitHub at [https://github.com/prevedel-lab/Deep3P-Denoising] and on Zenodo [https://doi.org/10.5281/zenodo.11175040].

For manuscripts utilizing custom algorithms or software that are central to the research but not yet described in published literature, software must be made available to editors and reviewers. We strongly encourage code deposition in a community repository (e.g. GitHub). See the Nature Portfolio guidelines for submitting code & software for further information.

Data

Policy information about availability of data

All manuscripts must include a data availability statement. This statement should provide the following information, where applicable:

- Accession codes, unique identifiers, or web links for publicly available datasets
- A description of any restrictions on data availability
- For clinical datasets or third party data, please ensure that the statement adheres to our policy

The source data are provided with this paper.

Research involving human participants, their data, or biological material

Policy information about studies with human participants or human data. See also policy information about sex, gender (identity/presentation), and sexual orientation and race, ethnicity and racism.

Reporting on sex and gender	We report the sex of donor patients for the cell lines. While we did not conduct sex-based analyses due to lower sample size which would not enable meaningful conclusions, the invasion patterns observed can be observed in both male and female patients. The two major cell lines analyzed are from a patient with female sex (S24) and male sex (T269). Gender was not assessed in this study.
Reporting on race, ethnicity, or other socially relevant groupings	No data regarding race, ethnicity or other socially relevant groupings was analyzed in this paper.
Population characteristics	-
Recruitment	-
Ethics oversight	Informed consent was obtained for all patients who donated their brain post-mortem to the Nervous System Tumor Bank. The protocol was reviewed and approved by the Northwestern University Institutional Review Board (IRB) under study STU00095863. All animal studies were performed with male NMRI nude mice up from an age of 8 weeks in accordance with the European Directive on animal experimentation (2010/63/EU) and institutional laboratory animal research guidelines after approval of the Regierungspräsidium Karlsruhe, Germany and the Animal Welfare Structure of the Luxembourg Institute of Health (protocol LRNO-2017-01). The 3R principles for reducing the number of animals were strictly followed and efforts were made to minimize animal suffering. Animals were scored daily and experiments were terminated in case of weight loss exceeding 10- 20%, neurological deficits and signs of pain, tumor size was no termination criterion. For all human tissues, patients have given informed consent and local regulatory authorities have approved (Ethic Committees at the Mannheim and Heidelberg Medical Faculty of the University Heidelberg, protocols (S-206/2005, S-207/2005, S-306/2019, 2018-614N-MA, 2018-843R-MA), the National Committee for Ethics in Research (CNER) Luxembourg (201201/06) and the regionale komiteer for medisinsk og helsefaglig forskningsetikk at the Helse Bergen (protocol 2013/720/REK vest). Molecular testing with IDH- R132H and ATRX stainings and the Illumina 850k methylation array (Department of Neuropathology, University of Heidelberg) for confirmation of diagnosis were performed.

Note that full information on the approval of the study protocol must also be provided in the manuscript.

Field-specific reporting

Please select the one below that is the best fit for your research. If you are not sure, read the appropriate sections before making your selection.

☒ Life sciences ☐ Behavioural & social sciences ☐ Ecological, evolutionary & environmental sciences

For a reference copy of the document with all sections, see nature.com/documents/nr-reporting-summary-flat.pdf

Life sciences study design

All studies must disclose on these points even when the disclosure is negative.

Sample size	Animal group sizes were as low as possible and empirically chosen and longitudinal measurements allowed a reduction of animal numbers by maintaining an adequate power. No statistical methods were used to predetermine sample size.
Data exclusions	Data was excluded in case of substantial registration errors or if data could not be analyzed.
Replication	Biological experiments were repeated with at least n = 3 per group.
Randomization	Image acquisition was based on tumor cell regions that showed tumor cell infiltration in the corpus callosum or deep in the cortex.
Blinding	Blinding during data acquisition was not necessary as e.g. groups of cells were acquired in the same field of view.

Behavioural & social sciences study design

All studies must disclose on these points even when the disclosure is negative.

Study description	*Briefly describe the study type including whether data are quantitative, qualitative, or mixed-methods (e.g. qualitative cross-sectional, quantitative experimental, mixed-methods case study).*
Research sample	*State the research sample (e.g. Harvard university undergraduates, villagers in rural India) and provide relevant demographic information (e.g. age, sex) and indicate whether the sample is representative. Provide a rationale for the study sample chosen. For studies involving existing datasets, please describe the dataset and source.*
Sampling strategy	*Describe the sampling procedure (e.g. random, snowball, stratified, convenience). Describe the statistical methods that were used to predetermine sample size OR if no sample-size calculation was performed, describe how sample sizes were chosen and provide a rationale for why these sample sizes are sufficient. For qualitative data, please indicate whether data saturation was considered, and what criteria were used to decide that no further sampling was needed.*
Data collection	*Provide details about the data collection procedure, including the instruments or devices used to record the data (e.g. pen and paper, computer, eye tracker, video or audio equipment) whether anyone was present besides the participant(s) and the researcher, and whether the researcher was blind to experimental condition and/or the study hypothesis during data collection.*
Timing	*Indicate the start and stop dates of data collection. If there is a gap between collection periods, state the dates for each sample cohort.*
Data exclusions	*If no data were excluded from the analyses, state so OR if data were excluded, provide the exact number of exclusions and the rationale behind them, indicating whether exclusion criteria were pre-established.*
Non-participation	*State how many participants dropped out/declined participation and the reason(s) given OR provide response rate OR state that no participants dropped out/declined participation.*
Randomization	*If participants were not allocated into experimental groups, state so OR describe how participants were allocated to groups, and if allocation was not random, describe how covariates were controlled.*

Ecological, evolutionary & environmental sciences study design

All studies must disclose on these points even when the disclosure is negative.

Study description	*Briefly describe the study. For quantitative data include treatment factors and interactions, design structure (e.g. factorial, nested, hierarchical), nature and number of experimental units and replicates.*
Research sample	*Describe the research sample (e.g. a group of tagged Passer domesticus, all Stenocereus thurberi within Organ Pipe Cactus National Monument), and provide a rationale for the sample choice. When relevant, describe the organism taxa, source, sex, age range and any manipulations. State what population the sample is meant to represent when applicable. For studies involving existing datasets, describe the data and its source.*
Sampling strategy	*Note the sampling procedure. Describe the statistical methods that were used to predetermine sample size OR if no sample-size calculation was performed, describe how sample sizes were chosen and provide a rationale for why these sample sizes are sufficient.*
Data collection	*Describe the data collection procedure, including who recorded the data and how.*
Timing and spatial scale	*Indicate the start and stop dates of data collection, noting the frequency and periodicity of sampling and providing a rationale for these choices. If there is a gap between collection periods, state the dates for each sample cohort. Specify the spatial scale from which the data are taken*
Data exclusions	*If no data were excluded from the analyses, state so OR if data were excluded, describe the exclusions and the rationale behind them, indicating whether exclusion criteria were pre-established.*
Reproducibility	*Describe the measures taken to verify the reproducibility of experimental findings. For each experiment, note whether any attempts to repeat the experiment failed OR state that all attempts to repeat the experiment were successful.*
Randomization	*Describe how samples/organisms/participants were allocated into groups. If allocation was not random, describe how covariates were controlled. If this is not relevant to your study, explain why.*
Blinding	*Describe the extent of blinding used during data acquisition and analysis. If blinding was not possible, describe why OR explain why blinding was not relevant to your study.*

Did the study involve field work? ☐ Yes ☐ No

Field work, collection and transport

Field conditions	*Describe the study conditions for field work, providing relevant parameters (e.g. temperature, rainfall).*
Location	*State the location of the sampling or experiment, providing relevant parameters (e.g. latitude and longitude, elevation, water depth).*
Access & import/export	*Describe the efforts you have made to access habitats and to collect and import/export your samples in a responsible manner and in compliance with local, national and international laws, noting any permits that were obtained (give the name of the issuing authority, the date of issue, and any identifying information).*
Disturbance	*Describe any disturbance caused by the study and how it was minimized.*

Reporting for specific materials, systems and methods

We require information from authors about some types of materials, experimental systems and methods used in many studies. Here, indicate whether each material, system or method listed is relevant to your study. If you are not sure if a list item applies to your research, read the appropriate section before selecting a response.

Materials & experimental systems

n/a	Involved in the study
☐	☒ Antibodies
☐	☒ Eukaryotic cell lines
☒	☐ Palaeontology and archaeology
☐	☒ Animals and other organisms
☐	☒ Clinical data
☒	☐ Dual use research of concern
☐	☒ Plants

Methods

n/a	Involved in the study
☒	☐ ChIP-seq
☒	☐ Flow cytometry
☐	☒ MRI-based neuroimaging

Antibodies

Antibodies used	Nestin antibody (abcam, Cat#ab6320, mouse monoclonal, clone 3k1, Lot number GR3415197-1, 1:500), Nestin (abcam, Cat#ab22035, mouse monoclonal, clone [102C], Lot numbers: GR3276045-9, GR312885-11, GR3404187-4, 1038495-2, 1038495-2, GR3404187-4, 1:100, 1:200, 1:500, 1:750), IDHR132H antibody (Dianova, Cat#DIAH09, mouse monoclonal, clone H09, 1:150, Lot Number: 21218/18), MBP antibody (Novus Cat# NB600-717, rat monoclonal, clone 12, Lot number: 153560, 1:100), MBP antibody (invitrogen, Cat#MA1-10837, mouse monoclonal, clone MBP101, Lot number: YI4054891, 1:100), Biotinylated anti-mouse antibody (abcam Cat#ab6788, goat polyclonal, Lot number: GR3213189-5, GR32455635-1, GR3245635-1,1:500), anti mouse antibody (Invtrogen, Cat#A11017, Lot numbers: 262519, 2626519,1:500), anti rat antibody (Invitrogen, Cat#A21247, Lot number: 1834715, 1:500)
Validation	Nestin antibodies: Venkataramani et al., Nature 2019, Yabo et al., Genome Med 2024.<br>IDH antibody: Capper et al., Acta Neuropathol., 2009.<br>MBP antibody: See specificity statement from manufacturer's website https://www.novusbio.com/PDFs/NB600-717.pdf and https://rnaidesigner.thermofisher.com/antibody/product/MBP-Antibody-clone-MBP101-Monoclonal/MA1-10837

Eukaryotic cell lines

Policy information about cell lines and Sex and Gender in Research

Cell line source(s)	A summary of all used cell lines including sex, age, and molecular characteristics can be found in Supplementary Table 2 and is not replicated here for reasons of space.
Authentication	Glioblastoma cell lines were authenticated with 850k methylation array analysis and subsequent classification.
Mycoplasma contamination	Cell lines are regularly tested for mycoplasma contamination.
Commonly misidentified lines (See ICLAC register)	-

Palaeontology and Archaeology

Specimen provenance	*Provide provenance information for specimens and describe permits that were obtained for the work (including the name of the issuing authority, the date of issue, and any identifying information). Permits should encompass collection and, where applicable, export.*
Specimen deposition	*Indicate where the specimens have been deposited to permit free access by other researchers.*
Dating methods	*If new dates are provided, describe how they were obtained (e.g. collection, storage, sample pretreatment and measurement), where they were obtained (i.e. lab name), the calibration program and the protocol for quality assurance OR state that no new dates are provided.*

☐ Tick this box to confirm that the raw and calibrated dates are available in the paper or in Supplementary Information.

Ethics oversight	*Identify the organization(s) that approved or provided guidance on the study protocol, OR state that no ethical approval or guidance was required and explain why not.*

Note that full information on the approval of the study protocol must also be provided in the manuscript.

Animals and other research organisms

Policy information about studies involving animals; ARRIVE guidelines recommended for reporting animal research, and Sex and Gender in Research

Laboratory animals	All animal studies were performed with male NMRI nude mice up from an age of 8 weeks.
Wild animals	No wild animals were used.
Reporting on sex	We used male nude mice for our patient-derived xenograft models as our experience has shown that male mice are more robust to perioperative risks during chronic window and tumor implantation. However, we made use of patient-derived xenograft lines that were derived from male and female glioblastoma patients. Therefore, we believe that the results from this project will be applicable to both male and female glioblastoma patients. Patient material from both male and female glioblastoma patients was analyzed.
Field-collected samples	-
Ethics oversight	In accordance with the European Directive on animal experimentation (2010/63/EU) and institutional laboratory animal research guidelines after approval of the Regierungspräsidium Karlsruhe, Germany, the EMBL IACUC, and the Animal Welfare Structure of the Luxembourg Institute of Health (protocol LRNO-2017-01). The 3R principles for reducing the number of animals were strictly followed and efforts were made to minimize animal suffering. Animals were scored daily and experiments were terminated in case of weight loss exceeding 10- 20%, neurological deficits and signs of pain, tumor size was no termination criterion.

Note that full information on the approval of the study protocol must also be provided in the manuscript.

Clinical data

Policy information about clinical studies

All manuscripts should comply with the ICMJEguidelines for publication of clinical research and a completedCONSORT checklist must be included with all submissions.

Clinical trial registration	Clinical trial was performed as described in Drumm et al., Neurooncology 2020.
Study protocol	Clinical trial was performed as described in Drumm et al., Neurooncology 2020.
Data collection	Clinical trial was performed as described in Drumm et al., Neurooncology 2020.
Outcomes	Clinical trial was performed as described in Drumm et al., Neurooncology 2020.

Dual use research of concern

Policy information about dual use research of concern

Hazards

Could the accidental, deliberate or reckless misuse of agents or technologies generated in the work, or the application of information presented in the manuscript, pose a threat to:

No	Yes	
☐	☐	Public health
☐	☐	National security
☐	☐	Crops and/or livestock
☐	☐	Ecosystems
☐	☐	Any other significant area

Experiments of concern

Does the work involve any of these experiments of concern:

No	Yes	
☐	☐	Demonstrate how to render a vaccine ineffective
☐	☐	Confer resistance to therapeutically useful antibiotics or antiviral agents
☐	☐	Enhance the virulence of a pathogen or render a nonpathogen virulent
☐	☐	Increase transmissibility of a pathogen
☐	☐	Alter the host range of a pathogen
☐	☐	Enable evasion of diagnostic/detection modalities
☐	☐	Enable the weaponization of a biological agent or toxin
☐	☐	Any other potentially harmful combination of experiments and agents

Plants

Seed stocks	-
Novel plant genotypes	-
Authentication	-

ChIP-seq

Data deposition

☐ Confirm that both raw and final processed data have been deposited in a public database such as GEO.

☐ Confirm that you have deposited or provided access to graph files (e.g. BED files) for the called peaks.

Data access links<br>*May remain private before publication.*	*For "Initial submission" or "Revised version" documents, provide reviewer access links. For your "Final submission" document, provide a link to the deposited data.*
Files in database submission	*Provide a list of all files available in the database submission.*
Genome browser session<br>(e.g. UCSC)	*Provide a link to an anonymized genome browser session for "Initial submission" and "Revised version" documents only, to enable peer review. Write "no longer applicable" for "Final submission" documents.*

Methodology

Replicates	*Describe the experimental replicates, specifying number, type and replicate agreement.*
Sequencing depth	*Describe the sequencing depth for each experiment, providing the total number of reads, uniquely mapped reads, length of reads and whether they were paired- or single-end.*
Antibodies	*Describe the antibodies used for the ChIP-seq experiments; as applicable, provide supplier name, catalog number, clone name, and lot number.*
Peak calling parameters	*Specify the command line program and parameters used for read mapping and peak calling, including the ChIP, control and index files used.*

Data quality	*Describe the methods used to ensure data quality in full detail, including how many peaks are at FDR 5% and above 5-fold enrichment.*
Software	*Describe the software used to collect and analyze the ChIP-seq data. For custom code that has been deposited into a community repository, provide accession details.*

Flow Cytometry

Plots

Confirm that:

- [ ] The axis labels state the marker and fluorochrome used (e.g. CD4-FITC).
- [ ] The axis scales are clearly visible. Include numbers along axes only for bottom left plot of group (a 'group' is an analysis of identical markers).
- [ ] All plots are contour plots with outliers or pseudocolor plots.
- [ ] A numerical value for number of cells or percentage (with statistics) is provided.

Methodology

Sample preparation	Glioma spheroid models were made into single cell suspesion using Accutase (Fisher scientific, A1110501). Glioblastoma cells were FACS sorted depending on their fluorescent mGFP signals. The filter used for GFP was 530/30.
Instrument	FACSAria Fusion 2 Bernhard Shoor BD<br>FACSAria Fusion Richard Sweet BD
Software	Data collection: FACS Diva Software<br>Data analysis: Flow Jo Version 10.5.x.
Cell population abundance	mGFP- positive S24 and T269 GBM cells were >95% positive post sort.
Gating strategy	Fluorescence minus one was used to define boundaries between positive and negative cells.

- [x] Tick this box to confirm that a figure exemplifying the gating strategy is provided in the Supplementary Information.

Magnetic resonance imaging

Experimental design

Design type	We did not perform fMRI, but DTI analysis.
Design specifications	-
Behavioral performance measures	-

Acquisition

Imaging type(s)	Diffusion Tensor Imaging (animal), T1 mpRAGE 3D and Diffusion Imaging, Resolve 2D sequence (human)
Field strength	9.4T (animal) and 3T (human MRI)
Sequence & imaging parameters	DTI: spin echo, field of view: 15x20 mm^2, acquisition matrix: 75x100, slice thickness: 0.5 mm, axial, TE: 17.57 ms, TR: 1000 ms<br>T1 mpRAGE 3D Sequence: TR: 1.7ms, TE: 3.3ms; FOV: 320x263 matrix size: 320x263 voxel size: 1mm. Diffusion imaging (Resolve 2D sequence): TR: 3.7ms, TE: 69ms, matrix size: 192x192 FOV: 220x220; slice thickness 5mm.
Area of acquisition	Whole brain scan was used.

Diffusion MRI [x] Used [ ] Not used

Preprocessing

Preprocessing software	Fractional Anisotropy (FA), Mean Diffusivity (MD) and Axial Diffusivity (AD) maps were extracted from the respective DTI image stacks using open-source software MITK-Diffusion (https://github.com/MIC-DKFZ/MITK-Diffusion/). Tracts of the body of the corpus callosum (CC) were extracted using the atTRACTive tool in MITK Diffusion.<br>(ADC) was quantified on a PACS RA1000 workstation (GE Healthcare, Chicago, US)
Normalization	We registered the images to a group standardized atlas.

Normalization template	Images underwent rigid registration within the Allen Mouse Brain Atlas employing MitkDReg.
Noise and artifact removal	No noise and artifact removal.
Volume censoring	No volume censoring.

Statistical modeling & inference

Model type and settings	n/a
Effect(s) tested	n/a

Specify type of analysis: ☐ Whole brain ☒ ROI-based ☐ Both

Statistic type for inference (See Eklund et al. 2016)	n/a
Correction	n/a

Models & analysis

n/a	Involved in the study
☒	☐ Functional and/or effective connectivity
☒	☐ Graph analysis
☒	☐ Multivariate modeling or predictive analysis

Functional and/or effective connectivity	*Report the measures of dependence used and the model details (e.g. Pearson correlation, partial correlation, mutual information).*
Graph analysis	*Report the dependent variable and connectivity measure, specifying weighted graph or binarized graph, subject- or group-level, and the global and/or node summaries used (e.g. clustering coefficient, efficiency, etc.).*
Multivariate modeling and predictive analysis	*Specify independent variables, features extraction and dimension reduction, model, training and evaluation metrics.*
